# Structured Cognitive-Motor Dual Task Training Compared to Single Mobility Training in Persons with Multiple Sclerosis, a Multicenter RCT

**DOI:** 10.3390/jcm8122177

**Published:** 2019-12-10

**Authors:** Renee Veldkamp, Ilse Baert, Alon Kalron, Andrea Tacchino, Mieke D’hooge, Ellen Vanzeir, Fanny Van Geel, Joke Raats, Mieke Goetschalckx, Giampaolo Brichetto, Nov Shalmoni, Peter Hellinckx, Natasja De Weerdt, Dorien De Wilde, Peter Feys

**Affiliations:** 1 REVAL Rehabilitation Research Center, Faculty of Rehabilitation Sciences, Hasselt University, 3500 Hasselt, BelgiumPeter.feys@uhasselt.be (P.F.); 2 Department of Physical Therapy, School of Health Professions, Sackler Faculty of Medicine, Tel-Aviv University, Ramat Aviv 6997801, Tel-Aviv, Israel; 3 Scientific Research Area, Italian MS Foundation (FISM), 16149 Genoa, Italy; 4 National MS Center Melsbroek, 1820 Steenokkerzeel, Belgium; 5 Rehabilitation and MS Center Overpelt, 3900 Pelt, Belgium; 6 De Mick AZ Klina, 2930 Brasschaat, Belgium; 7 AISM Rehabilitation Service of Genoa, Italian Multiple Sclerosis Society, 16149 Genoa, Italy; 8 Multiple Sclerosis Center, Sheba Medical Center, Derech Sheba 2, Ramat Gan 5262100, Tel-hashomer, Israel

**Keywords:** multiple sclerosis, cognitive motor interference, dual task, gait, balance, cognition

## Abstract

The aim was to compare the effectiveness of dual-task training (DTT) compared to single mobility training (SMT) on dual-task walking, mobility and cognition, in persons with Multiple Sclerosis (pwMS). Forty pwMS were randomly assigned to the DTT or SMT groups. The DTT-group performed dual-task exercises using an interactive tablet-based application, while the SMT-group received conventional walking and balance exercises. Both interventions were supervised and identical in weeks (8) and sessions (20). Nine cognitive-motor dual-task conditions were assessed at baseline, after intervention and at 4-weeks follow-up (FU). The dual-task cost (DTC), percentage change of dual-task performance compared to single-task performance, was the primary outcome. Mobility and cognition were secondarily assessed. Mixed model analyses were done with group, time and the interaction between group and time as fixed factors and participants as random factors. Significant time by group interactions were found for the digit-span walk and subtraction walk dual-task conditions, with a reduction in DTC (gait speed) for the DTT maintained at FU. Further, absolute dual-task gait speed during walking over obstacles only improved after the DTT. Significant improvements were found for both groups in various motor and cognitive measures. However, the DTT led to better dual-task walking compared to the SMT.

## 1. Introduction

Multiple sclerosis (MS) is characterized by both motor and cognitive impairments with a prevalence above 40% in both domains [1,2,3,4,5]. These impairments negatively impact quality of life. Moreover, in daily life the performance of a motor and a cognitive task simultaneously, such as walking while talking, is a common act. In people with MS (pwMS), the combination of walking and a cognitive task often leads to reduced performance in one or both tasks, called cognitive-motor interference (CMI) [6,7,8]. This CMI is often quantified by the dual task cost (DTC), which is the percentage of change in dual task performance relative to single-task performance [6]. Difficulties with cognitive-motor dual tasking have been related to higher risks of falls and lower quality of life in MS [9,10], this highlights the importance for rehabilitation strategies targeting CMI.

Different approaches have been proposed on how training might improve dual task performance [11]. One model focuses on automatization of an individual task, thereby reducing attentional requirements [12]. Alternatively, the task-integration model suggests that an integration of the two tasks is crucial for improvement and therefore tasks need to be practiced simultaneously in a dual task training (DTT) [12]. DTT-programs often try to incorporate cognitive components in a physical therapy program [13]. Important is that the motor task and the cognitive task are performed at the same time. Promising results for improving CMI with DTT in individuals with neurological disorders and elderly have been reported [13,14,15]. In MS, significant improvements in dual task performances after virtual reality treadmill training and exergames have been reported [13]. However, one may question the clinical utility of these technologies.

Only two studies on DTT compared to single task training have been conducted in pwMS. Sosnoff et al. reported a trend for greater gait speed under dual task conditions after a DTT compared to a single task training [16], while Monjezi et al. reported similar improvements in dual task walking after both types of training [17]. However, neither study examined the effects on the DTC and the Sosnoff et al. study was a feasibility study with a small sample size and no follow-up. Moreover, activities in daily life often include motor tasks like walking over obstacles or walking while carrying a cup. There is a need to examine the effects of a DTT that is standardized, individualized and progressive on a battery of cognitive-motor dual tasks with different complexities. Therefore, the aim of this randomized controlled trial is to investigate the effects of an integrated DTT compared to a single mobility training (SMT) on CMI in pwMS during various cognitive-motor dual tasks. It is hypothesized that the DTT will have a larger effect on dual task performance and on cognitive functioning than the SMT, while mobility will improve in both groups.

## 2. Experimental Section

### 2.1. Participants

Between November 2016 and June 2018, pwMS were recruited at the Rehabilitation and Multiple Sclerosis Centre Overpelt; National Multiple Sclerosis Center Melsbroek; AZ Klina, campus De Mick, Rehabilitation Brasschaat in Belgium; Italian Multiple Sclerosis Society (AISM) Rehabilitation Service of Genoa in Italy, and the Multiple Sclerosis Center, Sheba Medical Center, Tel-hashomer in Israel. Inclusion criteria were: diagnosis of MS according to McDonald criteria, age between 18 and 65, Expanded Disability Status Scale (EDSS) ≥2 and ≤6 [18], Mini Mental State Examination (MMSE) ≥ 26 [19], no relapse within the last 30 days, no changes in immunomodulatory disease treatment and no corticoid-therapy within the last 50 days and self-reported presence of dual task interference (dual task screening list ≥1) [20]. Participants were excluded if they had other medical conditions interfering with mobility, MS-like syndromes such as neuromyelitis optica, or when participants were not able to understand instructions or had major problems with hearing or vision. The study has been approved by the Ethical Committee of CHU Liège, Belgium, as well as by all the local ethical committees of the participating centers and executed according to the Helsinki declaration (ClinicalTrials Identifier NCT04158063). All participants received written information and signed informed consent.

### 2.2. Study Design and Procedure

The study was a multicenter, randomized two-arm controlled trial consisting of an integrated dual task training (DTT) group and a single mobility training (SMT) group. Blind randomization was done through sealed envelopes by the study project coordinator, who was not involved in intervention delivery or assessment. Stratification was used to balance age, gender and disability level (EDSS). Outcome measures were collected at baseline, after the intervention (post) and 4 weeks after post-measurement (follow-up). The study was blinded for the primary outcome measure, but not systematically blinded for the secondary measures as these tests were performed in the conventional departments. Detailed instruction booklets on both the assessment and intervention procedures were provided for each participating center.

### 2.3. Intervention

Both training groups included 20 supervised, individual sessions over a duration of eight weeks. The duration of the sessions was approximately 45 min, with regular rest periods between exercises. Training was provided by either a trained physical therapist, occupational therapist or neuropsychologist specialized in neurological rehabilitation.

The SMT consisted of 21 different gait and dynamic balance exercises with multiple difficulty levels. Duration of the exercises ranged from 30 s to 2 min. Progression was adjusted according to the patient’s abilities and the therapist’s judgement. A detailed description of the exercises can be found in Table S1.

The content of the DTT has previously been described in detail [21] and can be found in Table S2. The DTT was delivered through CMI-APP, an app developed specifically for the needs of the present RCT in a cooperation between rehabilitation scientists, clinicians working in the field of MS, pwMS and computer scientists. Generally, it consisted of exercises combining walking or stepping on the spot, with 11 different cognitive tasks of three difficulty levels each (easy, moderate, hard). Auditory cognitive tasks could be combined with walking and visual cognitive tasks could be combined with stepping on the spot. All exercises had a duration of 2 minutes. Subjects were instructed to perform both tasks as good as possible, to allow variable-priority training [12]. Feedback on the cognitive and motor performance was given after each dual task exercise, respectively by means of the accuracy of answers and the number of steps taken measured with a pedometer. Together with safety and quality considerations, this feedback led to pro- or regression to a higher or lower difficulty level, to allow a standardized, individualized and progressive training.

### 2.4. Measures

Age, gender, disease duration since diagnosis, type of MS, disability level (EDSS) and use of walking aid were collected as descriptive measures. Primary outcome measures of CMI and secondary outcome measures of cognition, mobility and patient-reported outcomes on quality of life, fatigue and dual task in daily life were administered at all time-points and are described below.

#### 2.4.1. Primary Outcome Measures

Single cognitive, single motor- and integrated cognitive-motor dual tasks were performed. All tasks had a duration of one minute. The order of tests was randomized among participants by the CMI-APP. However, the sequence of the tasks was similar at all time-points for the participant self.

The cognitive and motor tasks performed have been described in detail by Veldkamp et al., ([22]. Briefly, the cognitive tasks were: 1) the titrated digit span backwards [23], 2) the serial seven subtraction test [24] and 3) the auditory vigilance with alphabet task. Performance on the cognitive tasks was quantified as ‘number of correct answers’. The motor tasks were: 1) walking at self-paced speed (‘walk’), 2) walking while carrying a cup filled with water (‘cup’) and 3) walking while stepping over obstacles (10 cm height and width) placed every 3 m (‘obstacles’). Walking was assessed with three wearable APDM sensors (APDM, Inc., Portland, OR, USA) placed on each foot and in the lower lumbar region) and the related Mobility Lab Software (2017, APDM, Inc., Portland, OR, USA) and the performance was quantified as ‘gait speed (m/s)’ [25]. Participants also performed a fourth motor task, namely walking crisscross. However, as a recent study showed that performance on the ‘crisscross’ condition quantified by ‘turning velocity’ could not be reliably measured in persons with MS [22], these conditions were excluded from analysis in the present study. Similarly, results on the cognitive tasks is only shortly reported upon.

The three cognitive (‘digit span’, ‘subtraction’, ‘vigilance’) and three included motor tasks (‘walk’, ‘cup’, ‘obstacles’) were also simultaneously performed in nine different dual task conditions, for example ‘walk subtraction’. Participants were instructed to perform both tasks at their best level to avoid task prioritization. Dual task performance was quantified as motor and cognitive DTC (DTC_motor_ and DTC_cognitive_, respectively), as well as absolute motor and cognitive dual task performance for the nine dual task conditions. A decrement in performance during cognitive-motor dual tasking gives a positive DTC while an improvement gives a negative DTC:

(1) $${\mathrm{DTC}}_{\mathrm{motor}}\;(\%)=\frac{\left(\mathrm{single}-\mathrm{task}\;\mathrm{motor}\;\mathrm{score}\right)-\left(\mathrm{dual}-\mathrm{task}\;\mathrm{motor}\;\mathrm{score}\right)}{\mathrm{single}-\mathrm{task}\;\mathrm{motor}\;\mathrm{score}}\times 100$$

(2) $${\mathrm{DTC}}_{\mathrm{cognitive}}\;(\%)=\frac{\left(\mathrm{single}-\mathrm{task}\;\mathrm{cognitive}\;\mathrm{score}\right)-\left(\mathrm{dual}-\mathrm{task}\;\mathrm{cognitive}\;\mathrm{score}\right)}{\mathrm{single}-\mathrm{task}\;\mathrm{cognitive}\;\mathrm{score}}\times 100$$

#### 2.4.2. Descriptive and Secondary Outcome Measures

The Brief Repeatable Battery of Neuropsychological Tests (BRB-NT) [26] was used to assess participants’ cognitive function. The BRB-NT contains five tests in different cognitive domains. The Selective Reminding Test (SRT) is a verbal memory test, that measures verbal learning and delayed recall through wordlist learning over six trials. SRT Long term was scored as the total amount of words recalled in two consecutive trials and SRT Consistent as the total amount of words consistently recalled on all subsequent trials. The 10/36 Spatial Recall Test (SPART) assesses visuospatial learning and delayed recall. In this test, a checkerboard pattern with ten items must be learned and reproduced over three trials. The score is the total amount of correctly placed checkers. The Symbol Digital Modalities Test (SDMT) is a test of information processing speed and sustained attention that involves pairing symbols to numbers. The score is the total amount of correct oral responses within 90 s. The Paced Auditory Serial Addition Test (PASAT) assesses sustained attention, working memory and information processing speed. In this test, 61 digits are auditory presented with an interstimulus interval of 3 (PASAT-3s) or 2 (PASAT-2s) s. The task is to add each number to the one immediately before. The number of correct answers is scored. Lastly, in Word List Generation (WLG), semantic word fluency is measured by naming as many words as possible belonging to a certain category within 90 s. At post-intervention and follow-up measurements, parallel versions of the SDMT and the PASAT were administered.

Motor function in walking and balance was assessed using the Timed 25 Foot-Walk (T25FW) [27], Timed-Up-and-Go (TUG) test [28], Dynamic Gait Index (DGI) [29], 2-minute walking test (2MWT) [30], Multiple Sclerosis Walking Scale-12 (MSWS-12) [31] and Falls Efficacy Scale (FES-I) [32].

The Multiple Sclerosis Impact Scale-29 (MSIS-29) [33] and the Modified Fatigue Impact Scale (MFIS) [34] were used to record participants’ perceived impact of MS on day-to-day life and perception of fatigue in physical, cognitive and psychosocial domains, respectively. For the MSWS-12 and MSIS-29 physical and psychological subscales a transformed score between 0 and 100 was calculated. The Dual Task Questionnaire (DTQ) of Evans et al. [35] asked how often participants experienced problems with dual tasks (e.g., needing to stop an activity to talk). 

Furthermore, adherence was measured as amount of training sessions followed and after each training session participants perceived exertion was assessed by the Borg 15-point Rating of Perceived Exertion Scale (RPE) [36]. The scale score ranges from 6 to 20, where 6 means "no exertion at all (rest)" and 20 means "maximal exertion". At the end of the intervention, the Intrinsic Motivation Inventory (IMI) was administered. The IMI is a 30-item questionnaire with multiple choice questions starting from 1 (‘’not at all true’’) to 7 (‘’very true’’). The IMI assesses a participant’s subjective experience of the training through six subscales, namely interest/enjoyment, perceived competence, effort/importance, felt pressure and tension, value/usefulness and perceived choice.

### 2.5. Statistical Analysis

Data analysis was performed with JMP Pro 14 (SAS Institute Inc., Cary, NC, USA). A per-protocol analysis was conducted. Linear mixed models were used to analyze continuous outcomes. Normality was checked by conditional residual plots and extreme values were excluded from analysis. For normal distributed data, intervention group (DTT vs. SMT), time (pre, post, follow-up) and the interaction between time and group were included as fixed factors. Participants were included as random factor. Influence of center was checked by including center as random factor and nesting participants within the center, but this was discarded from the model as it was not significant for any of the primary outcome measures. For non-normal distributed data, the Mann-Whitney U test was performed to compare groups at all time-points and the Wilcoxon Signed Rank test was performed to compare pre with post, pre with follow-up and post with follow-up for both groups separately. Two sided *P*-values were set at α level 0.05 and for multiple comparisons a Bonferroni-correction (α = α/#tests) was conducted, with correction for time * group interaction α < 006 and time α < 017.

Baseline characteristics of participants and experience of and adherence to the training-program was compared between the intervention groups using an independent t-test or Mann-Whitney U test for normally and non-normally distributed continuous data as checked by a Shapiro-Wilk test, respectively. For nominal data, the Fisher exact or Chi^2^ test was used to compare the groups. A separate analysis checked whether people who dropped out of the study differed from those who remained.

## 3. Results

**Figure 1:**
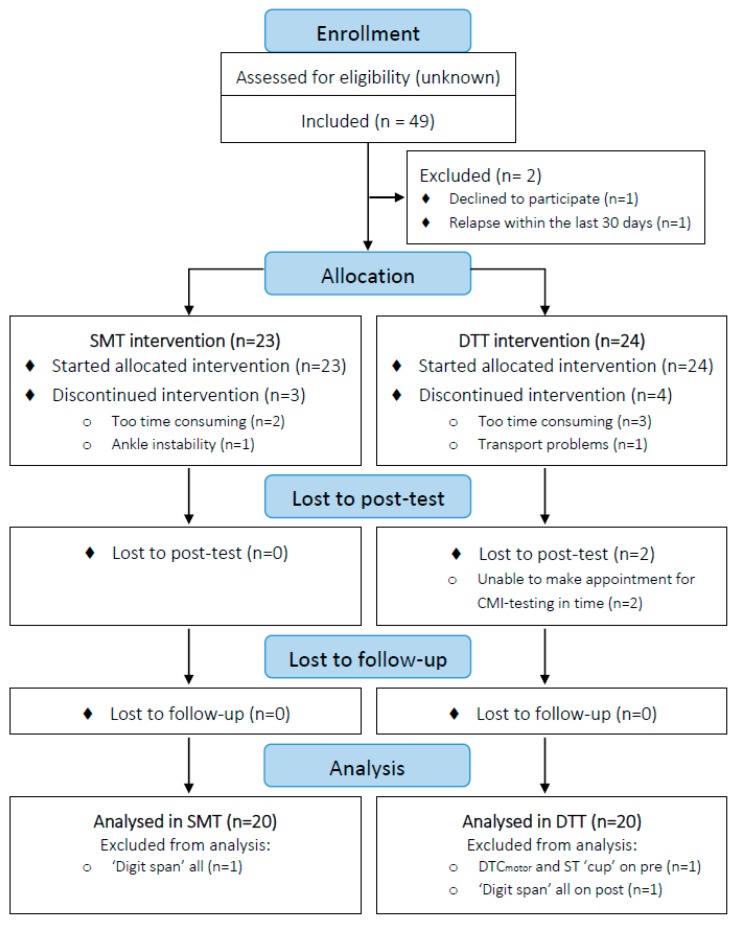
CONSORT Flow diagram.

The study flow is presented in Figure 1. In total, 16 persons from Belgium, 20 persons from Italy and 11 persons from Israel participated in the trial. Three persons dropped out of the SMT group and four persons dropped out of the DTT group, mostly due to time constraints (Figure 1). Ultimately, 20 participants per group were analyzed. Table 1 presents the descriptive characteristics of the participants per intervention group and for the dropouts. The participants who dropped out of the study were not significantly different from participants who completed the intervention, except for performance on the SRT test. At baseline, no significant differences were found between the DTT and SMT groups in any of the outcomes. No adverse events were reported in either group. During the intervention, the mean adherence was 94.3% and 95.8% for the DTT and SMT, respectively. No significant differences between groups were found in terms of perception of the training. On average, participants of both groups perceived the training as ‘’light to somewhat hard’’ as indicated by a mean RPE of 12.1 ± 1.7 for the DTT and 12.2 ± 2.7 for the SMT. For both interventions, participants generally enjoyed the training (IMI interest/enjoyment; DTT: 27.3 ± 5.4, SMT: 27.5 ± 5.8), felt competent (IMI perceived competence; DTT: 26.1 ± 5.1, SMT: 28.2 ± 4.0) and felt it was valuable (IMI value/usefulness; DTT: 30.5 ± 9.2, SMT: 34.6 ± 6.6), without feelings of pressure (IMI pressure/tension; DTT: 9.4 ± 4.8, SMT: 11.3 ± 6.0). They felt to some extent that the training was important and that they had a choice in performing the exercises and training (IMI effort/importance; DTT: 23.8 ± 7.6, SMT: 25.5 ± 7.0; IMI perceived choice; DTT: 19.6 ± 6.4, SMT: 20.1 ± 5.3).

### 3.1. Primary Outcome Measures

#### 3.1.1. Motor Task Performance in the Dual Task Conditions

**Figure 2:**
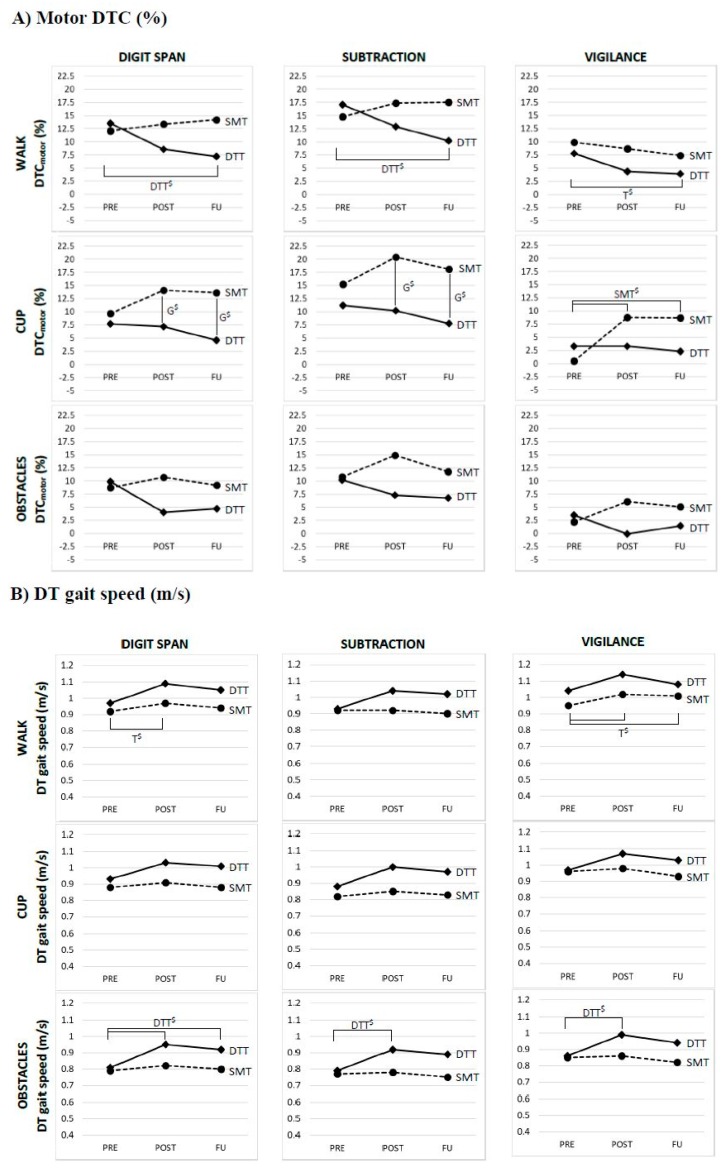
Graphs of the (**A**) DTC_motor_ and (**B**) absolute dual task gait speed (m/s) in the dual task conditions for the DTT (solid line) and SMT (dotted line). Post-hoc significant results are shown for time in both groups (T ^$^), group (G ^$^) or time*group interactions for DTT (DTT ^$^) or SMT (SMT ^$^). *Abbreviations:* DTC: Dual Task Cost; DT: Dual Task; DTT: Dual Task Training; SMT: Single Mobility Training.

The DTC_motor_ and dual task gait speed for the intervention groups on pre, post and follow-up, as well as the results of the mixed models analysis, are shown for all dual task conditions in Table 2. Figure 2 and Table 3 depict the results of the multiple comparisons. In total, 6.5% of the primary motor outcomes was not analyzed as a result of missing data, technical errors and an outlier.

For the DTC_motor_ a group*time interaction effect was found in the ‘walk digit span’ (*p* = 0.015), ‘walk subtraction’ (*p* = 0.006) and ‘cup vigilance’ (*p* = 0.013) conditions. Multiple comparisons showed a significant reduction of DTC_motor_ from pre to follow-up for the DTT in ‘walk digit span’ (*p* = 0.003) and ‘walk subtraction’ (*p* < 0.001) (Figure 2a). No significant effects were found for the SMT. In the ‘cup vigilance’ condition, multiple comparisons showed a significant increase of DTC_motor_ for SMT from pre to post (*p* <0.001) and from pre to follow-up (*p* < 0.001).

For the DTC_motor_ a significant main effect of time was found for ‘walk vigilance’ (*p* = 0.026), with a decrease in DTC_motor_ from pre to follow-up (*p* = 0.010). A significant main effect of group was found for DTC_motor_ in ‘cup digit span’ (*p* = 0.026) and ‘cup subtraction’ (*p* = 0.011). Multiple comparisons showed significant higher DTC_motor_ for SMT than DTT in ‘cup digit span’ and ‘cup subtraction’ on post (*p* = 0.042 and *p* = 0.009) and follow-up (*p* = 0.011 and *p* = 0.010). No significant effects were found for the DTC_motor_ in the ‘obstacles’ conditions.

For the absolute motor task performance during the dual task conditions, group*time interaction effects were found for dual task gait speed in the ‘obstacles digit span’ (*p* = 0.004), ‘obstacles subtraction’ (*p* = 0.031) and ‘obstacles vigilance’ (*p* = 0.006) conditions. Multiple comparisons showed a significant increase in dual task gait speed for the DTT-group in ‘obstacles digit span’ from pre to post (*p* < 0.001) and from pre to follow-up (*p* = 0.002), and from pre to post in the ‘obstacles subtraction’ (*p* = 0.003) and ‘obstacles vigilance’ (*p* < 0.001) conditions (Figure 2b). In all three ‘obstacles’ condition, this increased dual task gait speed was maintained at follow-up. No significant effects were found for the SMT-group.

Significant effect of time was found for dual task gait speed during ‘walk digit span’ (*p* = 0.021) and ‘walk vigilance’ (*p* = 0.019). A significant increase in dual task gait speed was shown after multiple comparisons during ‘walk digit span’ from pre to post (*p* = 0.009) and during ‘walk vigilance’ from pre to post (*p* = 0.013) and from pre to follow-up (*p* = 0.017). No significant effects were found for gait speed in dual ‘cup’ conditions. 

#### 3.1.2. Cognitive Task Performance in the Dual Task Conditions

The DTC_cognitive_, plus single and dual task performance on the cognitive task for the intervention groups on pre, post and follow-up are shown for the ‘digit span’, ‘subtraction’ and ‘vigilance’ conditions in Supplementary Tables S3, S4 and S5, respectively. In total, 3.3% of the primary cognitive outcomes was not analyzed as a result of missing data, technical errors and an outlier.

No group*time interaction effect nor a main effect of time or group was found for DTC_cognitive_ in any of the conditions. For the absolute number of correct answers on the digit span task, a significant main effect of time was found in the single digit span (*p* = 0.044) and ‘obstacles digit span’ (*p* = 0.003) conditions. Multiple comparisons showed a significant increase in number of correct answers from pre to post in those conditions (*p* = 0.013 and *p* = 0.0008, respectively). For number of correct answers on the subtraction task, a significant main effect of time was found in the single subtraction (*p* = 0.012), ‘walk subtraction’ (*p* < 0.0001) and ‘cup subtraction’ (*p* = 0.013) conditions. Multiple comparisons showed significant increase in performance from pre to follow up in all three of those subtraction conditions (*p* = 0.004, *p* < 0.0001, *p* = 0.016, respectively) and from pre to post for the subtraction walk and cup conditions (*p* < 0.0001 and *p* = 0.008, respectively).

The number of correct answers and DTC_cognitive_ on the vigilance task was not normally distributed. No significant differences between groups or between time-points within groups was found for any of the outcome measures.

### 3.2. Secondary Outcome Measures

Performance on the secondary outcomes on pre, post and follow-up as well as results of the mixed model analysis is shown in Table 4. In total, 1.4% of the data on secondary outcome measures was missing. No significant time*group interaction or main effect of group was found for any of the outcomes. Therefore, only main effects of time are shown in Table 4. A main effect of time was found for the T25FW (*p* = 0.006), TUG (*p* = 0.004), MSWS-12 (*p* = 0.033), DGI (*p* < 0.001), 2MWT (*p* = 0.033), SDMT (*p* = 0.023) and PASAT-3s (*p* < 0.001). Significant improvements from pre to post, which were maintained at follow-up, were found for T25FW, DGI and PASAT-3s. For the TUG, 2MWT and SDMT a significant improvement was found from pre to post, but this was not maintained at follow-up. Multiple comparisons showed no significant improvements on the MSWS-12. No significant effects were found for FES-I, DTQ, MSIS-29 subscales, MFIS and subscales and PASAT-2s.

## 4. Discussion

This study was the first multicenter, randomized controlled trial in MS that investigated the effects of a standardized, individualized, progressive integrated dual task training (DTT) compared to a single mobility training (SMT) on dual task performance in multiple dual task conditions relevant to daily life. The integrated DTT led to significant better improvements in dual task walking, including in more demanding dual tasks. Notably, these effects were found during untrained cognitive dual tasks and were retained at follow-up, indicating learning. Both, the DTT and the SMT, intervention groups showed improvements in dual task walking as shown by increased absolute dual task gait speed during the dual ‘walk’-conditions. This is consistent with earlier studies on DTT in MS, reporting similar findings between training-groups on absolute dual task gait speed while subtracting or alternating the alphabet, supporting the task automatization hypothesis [16,17].

This hypothesis states that you can reduce attentional capacity needed for a task by training a single task, resulting in automatization of this task according to traditional motor learning principles. This automatization of the motor task would result in increased dual task performance [12,37]. However, there were significant differences in impact of the interventions on dual task performances. Only the integrated DTT led to improved relative dual task performances when the cognitive task was more demanding, as shown by a decreased DTC_motor_ during walking while performing the digit span or subtraction task after the intervention. Additionally, only the DTT improved absolute dual task gait speed during walking over obstacles. These results favor the DTT over the SMT in improving dual task performance and are consistent with the task-integration hypothesis, stating that training integration of two tasks is crucial for improving dual task performance [12]. Together, these results suggest that both task automatization and task integration may play a role in improving dual task performance, but that integrated training is of added value in improving dual task walking in multiple dual task conditions in pwMS. Moreover, these improvements occurred without a decrease in cognitive dual task performance. Therefore, the improvements in dual task walking could not be explained by a tradeoff between the motor and cognitive tasks and thus reflect real improvements in dual task walking.

In contrast, our findings on dual task performances were not observed when participants had to walk with a filled cup. Although, this task is more complex than regular walking, it probably does not increase the risk of falling as much as when walking over obstacles. According to Yogev-Seligmann et al. prioritization strategies are an interplay between postural reserve, hazard estimation, and other factors in which in general people would focus on the cognitive task as long as the postural threat is relatively low [38]. The prioritization of tasks might have been more variable in the walking with cup conditions than in the usual walking or walking over obstacles conditions. Surprisingly, no improvements were found on patient-reported experiences of dual tasking in daily life as measured by the questionnaire. It might be that the effects found in the dual task measures were not big enough to be clinically relevant, as the changes in DTC_motor_ in the present study were lower than the previously reported minimal detectable change of approximately 12% for the DTC_motor_ [22]. Other explanations of the lack of improvement on the dual task questionnaire might be that the activities in daily life are still somehow different from the dual tasks used in the current study (e.g. unpredictable events or tasks with a longer duration), or the fact that the used dual task questionnaire has not been developed for or examined on validity or responsiveness in pwMS.

Contrary to the findings on the motor performances, we did not observe improvements on the DTC_cognitive_ for either group. Besides, both groups showed in some conditions improvements on the absolute cognitive performance of the ‘digit span’ and ‘subtraction’ tasks. According with literature, this issue is unclear. Monjezi et al. [17] reported increases in single and dual task performance on the cognitive task after both an integrated dual task and a single task intervention. Other studies however did find a difference between DTT and single task training on cognitive dual task performance in Parkinson disease patients [39] and elderly subjects [12]. Although the cognitive tasks used in the present study are conventional in literature, the DTC_cognitive_ recently demonstrated low reliability and learning effects in pwMS, perhaps explaining part of these results [22]. 

Furthermore, no differential effects of the intervention on the SDMT and PASAT were found, both groups showed improvements. This might be attributed to the fact that these are measures of information processing speed, while the cognitive tasks in the DTT didn’t specifically target cognitive speed. These results might also indicate that the cognitive exercises in the DTT did not reach a degree of complexity that was challenging enough to improve cognition or cognitive dual task performance.

Both groups performed better on various measures of mobility as the T25FW, TUG, DGI and 2MWT after intervention. The DTT increased distance walked on the 2MWT above the clinically meaningful change post-intervention from both patient (9.6 m) and therapist (6.8 m) perspectives [40], with 12.9 m post intervention and with 6.3m at follow-up. This was not true for the SMT group. In contrast, while not significant, the SMT did result in a decrease of 9.9 points on the MSWS-12 post intervention, which is close to the clinical meaningful change from patient and therapist perspectives (−10.4 and −11.4 points, respectively) [40]. The DTT did not. Thus, the DTT resulted in increased walking endurance and functional walking capacity, while the patient-rated walking ability seemed to improve clinically more after the SMT.

Methodologically, the use of a novel app-based tool, allowing for standardized assessment of a wide variety of dual task conditions and application of a structured, progressive intervention in a multicenter trial context, constitutes the strengths of the study. However, some limitations are acknowledged. Firstly, the motor part of both training groups was not identical, as the SMT included more diverse mobility exercises than only walking or stepping on the spot as in the DTT. However, no significant differences in improvements on mobility outcomes have been found and participants showed similar scores on measures of perceived exertion and intrinsic motivation. Those results also showed that concerns on whether the interventions would be too fatiguing or not motivating enough are not needed. Second, due to the multicenter, clinical set-up, the blinding of the secondary outcome measures was not always done. Secondary measures have often been collected by therapists of the center who may have known the group allocation. Third, it is not entirely known in which way the presence of the obstacles distorts the measurement of walking speed. However, in a test-retest reliability study in MS, absolute dual task gait speed while walking over obstacles was found to be excellent. Lastly, the inclusion criteria were not fully met for all participants as for one participant the score on the dual task Screening list was missing and two participants scored zero. However, as they all showed a DTC_motor_ higher than 10% during walking while subtraction sevens, it was decided to keep them in the analysis.

The participants in the present study were in general not severely motor or cognitive impaired and neither severely fatigued. The results are therefore not generalizable to more severely impaired pwMS. Patient characteristics as degree of motor or cognitive impairment, or fatigue might influence responsiveness to the different interventions. For example, severe cognitive impairment might reduce the possibility to gain improvement from the training because of cognitive overload [41]. In the current study, groups were too small to thoroughly analyze these possible effects. Therefore, a larger trial including persons with severe MS is needed to develop guidelines for an individualized DTT across disabilities.

## 5. Conclusions

This study showed beneficial effects of an integrated DTT compared to a SMT on dual task performances. The effects were mostly retained at follow-up, showing dual task motor learning in MS after a DTT. No adverse events occurred during the DTT. These promising results can guide therapists to include dual tasks in their rehabilitation programs for persons with MS. Future studies should seek to determine who benefits most from DTT.

## Figures and Tables

**Table 1:** Descriptive characteristics at baseline for DTT, SMT and drop-outs.

	DTT (*n* = 20)	SMT (*n* = 20)	*p*-value DTT vs. SMT	Drop-out (*n* = 7)	*p*-value Drop-out vs. Remained
Age (years)	51.4 ± 9.3	53.4 ± 9.2	0.507	49.5 ± 8.5	0.436
Gender (F/M, %)	60/40	55/45	1.00	85.7/14.3	0.225
Type of MS (RR/SP/PP%)	65/20/15	65/15/20	0.867	100/0/0 ^a^	0.221
EDSS (0–10) Min—Max	3.4 ± 1.0 2—5	3.7 ± 1.2 2—6	0.404	3.9 ± 1.1 ^a^	0.456
Years since diagnosis	9.6 ± 7.7	11.4 ± 9.8	0.627	12.4 ± 10.1 ^a^	0.525
Walking aid (yes/no, %)	35/65	15/85	0.273	16.7/83.3 ^a^	1.00
MMSE (0–30)	28.5 ± 1.3	28.8 ± 1.2	0.478	28.8 ± 1.8 ^a^	0.434
DT Screening list (0–13)	5.3 ± 2.7 ^a^	4.1 ± 3.3	0.218	4.6 ± 2.7 ^a^	0.949
SRT Long term (0–72)	40.1 ± 13.5	36.1 ± 17.1	0.412	50.9 ± 9.4	0.040 *
SRT Consistent (0–72)	29.8 ± 17.6	27.2 ± 17.4	0.635	38.0 ± 16.8	0.075
SRT Delay (0–12)	8.1 ± 2.8	7.9 ± 2.6	0.772	8.4 ± 3.9	0.702
SPART (0–30)	18.9 ± 6.2	18.3 ± 8.2	0.779 ^b^	22.4 ± 5.8	0.150
SPART Delay (0–10)	6.4 ± 2.5	6.6 ± 2.5	0.904	8.3 ± 2.1	0.092
WLG (*n*)	22.8 ± 5.1	23.2 ± 5.2	0.854	26.3 ± 8.4	0.239

Data are presented as mean ± SD or percentages (%). ^a^ Data for one person is missing, ^b^ Equal variances not assumed. Significant at < 0.05 (*). Abbreviations: DTT: Dual Task Training; SMT: Single Mobility Training; MS: Multiple Sclerosis; n: number; RR: Relapsing Remitting; SP: Secondary Progressive; PP: Primary Progressive; EDSS: Expanded Disability Status Scale; MMSE: Mini Mental State Examination; DT: Dual Task; SRT: Selective Reminding Test; SPART: 10/36 Spatial Recall Test; WLG: Word List Generation, SD: standard deviation.

**Table 2:** Means and mixed model analysis of DTC_motor_ during all Dual Task conditions.

	Group	Digit Span (mean ± SD)			Subtraction (mean ± SD)			Vigilance (mean ± SD)		
		Pre	Post	Fu	Pre	Post	Fu	Pre	Post	Fu
**Walk** **DTC_motor_ (%)**	DTT	13.5 ± 7.6 (*n* = 18)	8.6 ± 7.8 (*n* = 18)	7.2 ± 10.0 (*n* = 19)	17.1 ± 9.5 (*n* = 18)	12.9 ± 10.0 (*n* = 18)	10.2 ± 7.1 (*n* = 19)	7.8 ± 5.6 (*n* = 17)	4.4 ± 5.2 (*n* = 18)	3.9 ± 7.0 (*n* = 19)
	SMT	12.1 ± 10.5 (*n* = 19)	13.4 ± 9.5 (*n* = 18)	14.2 ± 8.6 (*n* = 19)	14.8 ± 8.3 (*n* = 18)	17.4 ± 8.8 (*n* = 18)	17.5 ± 9.5 (*n* = 19)	9.9 ± 9.7 (*n* = 19)	8.7 ± 6.5 (*n* = 18)	7.4 ± 6.7 (*n* = 19)
*p*-values		*T: 0.286*	*G: 0.171*	T*G: 0.015 *	*T: 0.129*	*G: 0.169*	T*G: 0.006*	T: 0.026*	*G: 0.090*	T*G: 0.699
**Walk** **Speed (m/s)**	DTT	0.97 ± 0.23 (*n* = 19)	1.09 ± 0.22 (*n* = 18)	1.05 ± 0.24 (*n* = 20)	0.93 ± 0.23 (*n* = 19)	1.04 ± 0.23 (*n* = 18)	1.02 ± 0.24 (*n* = 20)	1.04 ± 0.25 (*n* = 18)	1.14 ± 0.22 (*n* = 18)	1.08 ± 0.26 (*n* = 20)
	SMT	0.92 ± 0.26 (*n* = 20)	0.97 ± 0.24 (*n* = 18)	0.94 ± 0.23 (*n* = 20)	0.92 ± 0.25 (*n* = 18)	0.92 ± 0.23 (*n* = 18)	0.90 ± 0.24 (*n* = 20)	0.95 ± 0.26 (*n* = 20)	1.02 ± 0.23 (*n* = 18)	1.01 ± 0.25 (*n* = 20)
*p*-values		T: 0.021*	*G: 0.175*	T*G: 0.081	*T: 0.059*	*G: 0.158*	T*G: 0.077	T: 0.019 *	*G: 0.200*	T*G: 0.237
**Cup** **DTC_motor_ (%)**	DTT	7.7 ± 9.0 (*n* = 17)	7.2 ± 9.1 (*n* = 18)	4.6 ± 12.6 (*n* = 19)	11.2 ± 11.8 (*n* = 16)	10.1 ± 8.2 (*n* = 18)	7.8 ± 11.7 (*n* = 18)	3.3 ± 6.6 (*n* = 17)	3.3 ± 8.3 (*n* = 18)	2.3 ± 7.4 (*n* = 19)
	SMT	9.7 ± 14.7 (*n* = 19)	14.1 ± 9.0 (*n* = 18)	13.6 ± 6.3 (*n* = 19)	15.2 ± 17.0 (*n* = 19)	20.4 ± 10.5 (*n* = 18)	18.1 ± 8.0 (*n* = 19)	0.5 ± 11.3 (*n* = 19)	8.8 ± 8.4 (*n* = 18)	8.7 ± 6.5 (*n* = 19)
*p*-values		*T: 0.675*	G: 0.026 *	T*G: 0.227	*T: 0.501*	G: 0.011 *	T*G: 0.340	T: 0.033 *	*G: 0.126*	T*G: 0.013 *
**Cup** **Speed (m/s)**	DTT	0.93 ± 0.26 (*n* = 19)	1.03 ± 0.27 (*n* = 18)	1.01 ± 0.29 (*n* = 20)	0.88 ± 0.26 (*n* = 18)	1.00 ± 0.27 (*n* = 18)	0.97 ± 0.28 (*n* = 19)	0.97 ± 0.28 (*n* = 19)	1.07 ± 0.25 (*n* = 18)	1.03 ± 0.26 (*n* = 20)
	SMT	0.88 ± 0.26 (*n* = 20)	0.91 ± 0.24 (*n* = 18)	0.88 ± 0.26 (*n* = 20)	0.82 ± 0.25 (*n* = 20)	0.85 ± 0.24 (*n* = 18)	0.83 ± 0.26 (*n* = 20)	0.96 ± 0.26 (*n* = 20)	0.98 ± 0.28 (*n* = 18)	0.93 ± 0.27 (*n* = 20)
*p*-values		*T: 0.126*	*G: 0.164*	T*G: 0.115	*T: 0.113*	*G: 0.087*	T*G: 0.155	*T: 0.622*	*G: 0.347*	T*G: 0.101
**Obstacles** **DTC_motor_ (%)**	DTT	9.9 ± 9.9 (*n* = 17)	4.0 ± 7.7 (*n*=18)	4.7 ± 10.0 (*n* = 18)	10.2 ± 7.4 (*n* = 17)	7.3 ± 8.5 (*n* = 18)	6.8 ± 9.4 (*n* = 19)	3.5 ± 9.5 (*n* = 18)	−0.1 ± 5.1 (*n* = 18)	1.4 ± 8.1 (*n* = 19)
	SMT	8.7 ± 7.9 (*n* = 19)	10.7 ± 11.5 (*n* = 18)	9.2 ± 7.8 (*n* = 19)	10.8 ± 10.4 (*n* = 19)	14.9 ± 9.3 (*n* = 18)	11.8 ± 12.3 (*n* = 19)	2.2 ± 6.7 (*n* = 19)	6.1 ± 8.8 (*n* = 18)	5.1 ± 4.1 (*n* = 19)
*p*-values		*T: 0.329*	*G: 0.189*	T*G: 0.072	*T: 0.576*	*G: 0.090*	T*G: 0.211	*T: 0.957*	*G: 0.101*	T*G: 0.064
**Obstacles** **Speed (m/s)**	DTT	0.81 ± 0.24 (*n* = 18)	0.95 ± 0.22 (*n* = 18)	0.92 ± 0.24 (*n* = 19)	0.79 ± 0.21 (*n* = 18)	0.92 ± 0.23 (*n* = 18)	0.89 ± 0.24 (*n* = 20)	0.86 ± 0.26 (*n* = 19)	0.99 ± 0.23 (*n* = 18)	0.94 ± 0.25 (*n* = 20)
	SMT	0.79 ± 0.26 (*n* = 20)	0.82 ± 0.22 (*n* = 18)	0.80 ± 0.23 (*n* = 19)	0.77 ± 0.24 (*n* = 20)	0.78 ± 0.22 (*n* = 18)	0.75 ± 0.24 (*n* = 20)	0.85 ± 0.25 (*n* = 20)	0.86 ± 0.23 (*n* = 18)	0.82 ± 0.24 (*n* = 20)
*p*-values		T: 0.017*	*G: 0.162*	T*G: 0.004*	*T: 0.156*	*G: 0.100*	T*G: 0.031*	*T: 0.105*	*G: 0.179*	T*G: 0.006 *

Significant at < 0.05 (*). Abbreviations: DTT: dual task training; SMT: single mobility training; DTC: dual task cost; FU: follow-up; T: time; G: group; T*G: interaction term.

**Table 3:** Multiple comparisons DTC_motor_ and Dual Task gait speed.

**DTC_motor_ (%)**	**Effect of**	**Post-hoc analysis *p*-values ^a^**		
Walk-Digit Span	Time*Group	DTT_PRE-POST_: 0.022 SMT_PRE-POST_: - PRE_DTT-SMT_: -	DTT_PRE-FU_: 0.003 ^$^ SMT_PRE-FU_: - POST_DTT-SMT_: -	DTT_POST-FU_: - SMT_POST-FU_: - FU_DTT-SMT_: 0.019
Walk-Subtraction	Time*Group	DTT_PRE-POST_: 0.024 SMT_PRE-POST_: - PRE_DTT-SMT_: -	DTT_PRE-FU_: <0.001 ^$^ SMT_PRE-FU_: - POST_DTT-SMT_: -	DTT_POST-FU_: - SMT_POST-FU_: - FU_DTT-SMT_: 0.017
Walk-Vigilance	Time	PRE-POST: 0.043	PRE-FU: 0.010 ^$^	POST-FU: -
Cup-Digit Span	Group	PRE_DTT-SMT:_ -	POST_DTT-SMT_: 0.042 ^$^	FU_DTT-SMT_: 0.011 ^$^
Cup-Subtraction	Group	PRE_DTT-SMT:_ -	POST_DTT-SMT_: 0.009 ^$^	FU_DTT-SMT_: 0.010 ^$^
Cup-Vigilance	Time*Group	DTT_PRE-POST_: - SMT_PRE-POST_:<0.001 ^$^ PRE_DTT-SMT_: -	DTT_PRE-FU_: - SMT_PRE-FU_: <0.001 ^$^ POST_DTT-SMT_: 0.043	DTT_POST-FU_: - SMT_POST-FU_: - FU_DTT-SMT_: 0.021
**DT gait speed (m/s)**	**Effect of**	**Post-hoc analysis *p*-values ^a^**		
Walk-Digit Span	Time	PRE-POST: 0.009 ^$^	PRE-FU: 0.034	POST-FU: -
Walk-Vigilance	Time	PRE-POST: 0.013 ^$^	PRE-FU: 0.017 ^$^	POST-FU: -
Obstacles-Digit Span	Time*Group	DTT_PRE-POST_: <0.001 ^$^ SMT_PRE-POST_: - PRE_DTT-SMT_: -	DTT_PRE-FU_: 0.002 ^$^ SMT_PRE-FU_: - POST_DTT-SMT_: 0.040	DTT_POST-FU_: - SMT_POST-FU_: - FU_DTT-SMT_: -
Obstacles-Subtraction	Time*Group	DTT_PRE-POST_: 0.003 ^$^ SMT_PRE-POST_: - PRE_DTT-SMT_: -	DTT_PRE-FU_: 0.023 SMT_PRE-FU_: - POST_DTT-SMT_: 0.033	DTT_POST-FU_: - SMT_POST-FU_: - FU_DTT-SMT_: 0.069
Obstacles-Vigilance	Time*Group	DTT_PRE-POST_: <0.001 ^$^ SMT_PRE-POST_: - PRE_DTT-SMT_: -	DTT_PRE-FU_: 0.026 SMT_PRE-FU_: - POST_DTT-SMT_: 0.054	DTT_POST-FU_: - SMT_POST-FU_: - FU_DTT-SMT_: -

Significant at post-hoc (^$^). ^a^ *p*-values < 0.10 are shown; -: *p*-value > 0.10. Abbreviations: DTT: dual task training; SMT: single mobility training; DTC: dual task cost; DT: dual task; FU: follow-up.

**Table 4:** Means and mixed model analysis of secondary outcome measures.

	Dtt			Smt			*p*-value	Multiple Comparisons *p*-value		
	Pre	Post	Fu	Pre	Post	Fu	Time ^a^	Pre-post	Pre-fu	Post-fu
**Mobility** (mean ± SD)										
T25FW (s)	6.2 ± 1.5	5.3 ± 1.2	5.5 ± 1.4	6.4 ± 2.6	6.0 ± 2.5	5.7 ± 1.8	0.006 *	0.010 ^$^	0.003 ^$^	0.649
TUG (s)	8.6 ± 2.2	7.6 ± 1.7	8.3 ± 2.6	8.8 ± 3.3	8.0 ± 2.7	8.5 ± 2.8	0.004 *	0.001 ^$^	0.204	0.036
MSWS-12 (0–100)	37.6 ± 21.4	36.6 ± 23.0	32.2 ± 22.2	40.7 ± 23.9	30.8 ± 26.6	34.0 ± 29.3	0.033 *	0.031	0.018	0.846
DGI (0–24)	19.2 ± 4.3	21.1 ± 2.6	21.9 ± 2.1	20.3 ± 3.2	21.4 ± 3.5	21.7 ± 2.9	<0.001 *	<0.001 ^$^	<0.001 ^$^	0.129
FES-I (16–64)	30.9 ± 11.7	28.2 ± 8.6	27.7 ± 9.0	28.3 ± 9.5	26.8 ± 9.4	29.1 ± 10.6	0.224			
2MWT (m)	144.1 ± 42.1	157.0± 33.6	150.4± 45.6	141.1± 37.1	147.9± 40.3	143.9± 39.4	0.033 *	0.010 ^$^	0.135	0.247
**Cognitive** (mean ± SD)										
SDMT (0–110)	46.8 ± 11.6	48.8 ± 14.7	46.0 ± 14.7	44.7 ± 12.2	48.2 ± 10.5	45.5 ± 10.3	0.023 *	0.017 ^$^	1.00	0.017 ^$^
PASAT-3s (0–60)	42.2 ± 12.8	47.4 ± 12.0	48.2 ± 12.4	46.9 ± 9.8	49.4 ± 7.7	49.2 ± 12.4	<0.001 *	0.001 ^$^	<0.001 ^$^	0.927
PASAT-2s (0–60)	34.0 ± 12.1	37.7 ± 12.6	40.2 ± 12.5	40.0 ± 11.5	41.0 ± 11.7	41.6 ± 11.6	0.078		-	
**Patient-reported** (mean ± SD)										
DTQ (0–40)	13.8 ± 8.7	11.8 ± 6.8	12.5 ± 9.0	13.0 ± 9.1	11.8 ± 6.9	12.15 ± 7.6	0.154	-		
MSIS-29 Phys (0–100)	30.4 ± 21.9	27.6 ± 18.6	26.4 ± 20.7	26.0 ± 17.8	22.9 ± 16.8	24.6 ± 19.2	0.106			
MSIS-29 Psycho (0–100)	29.7 ± 21.3	28.5 ± 23.4	28.9 ± 23.6	33.2 ± 24.3	29.0 ± 21.7	31.5 ± 20.3	0.416			
MFIS Total (0–84)	35.5 ± 19.0	34.7 ± 20.4	34.5 ± 20.3	29.8 ± 19.1	27.8 ± 18.7	28.0 ± 18.2	0.403			
MFIS Phys (0–36)	17.1 ± 8.5	16.8 ± 9.0	16.6 ± 9.8	14.5 ± 8.6	13.8 ± 8.5	13.9 ± 8.9	0.739			
MFIS Psycho (0–8)	3.1 ± 2.4	3.0 ± 2.6	3.2 ± 2.6	2.2 ± 2.3	2.2 ± 2.2	2.0 ± 2.2	0.805			
MFIS Cognitive (0–40)	15.4 ± 10.9	14.8 ± 10.5	14.8 ± 9.9	13.2 ± 9.8	11.9 ± 9.7	12.2 ± 9.1	0.247			

^a^ Only main effects of time are shown, as there were no significant effects of group or time*group. Significant at < 0.05 (*), significant post-hoc (^$^). Abbreviations: DTT: Dual Task Training; SMT: Single Mobility Training; FU; follow-up; T25FW: Timed 25 Foot-Walk; TUG: Timed-Up-and-Go; MSWS-12: Multiple Sclerosis Walking Scale-12; DGI: Dynamic Gait Index; FES-I: Falls Efficacy Scale-International; 2MWT: 2-Minute Walking Test; SDMT: Symbol Digital Modalities Test; PASAT: Paced Auditory Serial Addition Test; DTQ: Dual Task Questionnaire; MSIS-29: Multiple Sclerosis Impact Scale-29; phys: physical subscale; psycho: psychological subscale; MFIS: Modified Fatigue Impact Scale, SD: standard deviation.

## Supplementary Materials

The following are available online at https://www.mdpi.com/2077-0383/8/12/2177/s1, Table S1: Exercises during SMT. Table S2: Exercises during DTT. Table S3: Means and mixed model analysis of cognitive DTC and correct answers (#n) in ‘Digit Span’ conditions. Table S4: Means and mixed model analysis of cognitive DTC and correct answers (#n) in ‘Subtraction’ conditions. Table S5: Means and statistical analysis of cognitive DTC and correct answers (#n) in ‘Vigilance’ conditions.
