# Supplementary material for: Structured Cognitive-Motor Dual Task Training Compared to Single Mobility Training in Persons with Multiple Sclerosis, a Multicenter RCT
Source: J Clin Med. 2019 Dec 10;8(12):2177. doi: 10.3390/jcm8122177 (PMC6947084; doi:10.3390/jcm8122177)
Supplement: Supplementary file 1 [file jcm-08-02177-s001.pdf]

Table S1; Exercises during SMT.

Exercise	Exercise description / instruction	Difficulty levels
**Normal gait speed**	Walk at your normal speed	1. for 2 min<br>2. over uneven underground for 2 min<br>3. backwards for 1 min
**Fast gait speed**	Walk as fast as you can (not running)	1. for 30sec<br>2. for 60sec<br>3. for 90sec
**Running**	Running without limping.	1. for 15sec, 5*, ± 10sec breaks between<br>2. for 30sec, 3*, ± 10sec breaks between<br>3. for 60sec, 2*, ± 10sec breaks between
**Gait quality**	Depending on clinical need: walking while focus on heel strike, knee raise and/or hip flexion	1. for 2 min<br>2. for 2 min, increase amplitude<br>3. for 2 min, increase speed
**Stepping on the spot**	Step on the spot or on and off a bench.	1. stepping on the spot for 2 min<br>2. march up and down on a 15cm step for 2 min<br>3. march up and down on a 30cm step, for 2 min
**Stepping**	Stepping forwards, backwards, to the right, to the left.	1. step sideways to the left and left alternately over a cane, for 2 min<br>2. step forwards and backwards alternately over a cane, for 2 min<br>3. step forwards, to the right, backwards, to the left and counterclockwise back over canes, for 2 min
**Change in gait speed**	Begin walking at your normal pace. When I tell you "Go", walk as fast as you can. When I tell you "Slow", walk as slowly as you can.	1. for 2 min with fixed presentation interval (every 30sec) and execution time (5sec)<br>2. for 2 min with variable presentation interval (10-30sec) and execution time (10sec)<br>3. for 3 min with variable presentation interval (10-30sec) and execution time (10sec)
**Gait with horizontal head turning**	Begin walking at your normal pace. When I tell you "Look right", keep walking straight, but turn your head to the right. Keep looking to the right until I tell you, "Look left". Then keep walking straight and turn your head to the left until I tell you "Look straight". Where after you keep walking straight, but return your head to the center.	1. While standing, turn to look directly behind you toward left shoulder for 10sec. Repeat to the right. For 2 min<br>2. While walking, every 15 sec head turn, for 2 min<br>3. While walking, every 10 sec head turn, for 2 min
**Gait with vertical head turning**	Begin walking at your normal pace. When I tell you "Look up", keep walking straight, but tip your head up. Keep looking up until I tell you, "Look down". Then keep walking straight and tip your head down. Keep your head down until I tell you "Look straight", then keep walking straight, but return your head to the center.	1. While standing, turn to look up for 10sec. Repeat to look down. For 2 min<br>2. While walking, every 15 sec head up-down, for 2 min<br>3. While walking, every 10 sec head up-down, for 2 min
**Pivot turning**	Begin walking at your normal pace. When I tell you "Turn and stop", turn as quickly as you can to face the opposite direction and stop.	1. 3 turns at 15sec, at 30sec and at 45sec, for 1 min<br>2. 6 turns at 10, 30, 50, 70, 90, 110 sec, for 2 min<br>3. 6 turns with a variable time interval, for 2 min
**Reaching forwards**	The patient is standing at a distance from a wall with a poster with 9 numbers (40*40cm squares) so that number 5 is on the level of the patient's sternum and the patient can reach comfortably the numbers without trunk movements or strong elevation of the shoulders. The therapist says which number (at random) the patient needs to touch with his preferable hand.	1. both feet on the floor, for 2 min<br>2. both feet on balance foam, for 2 min<br>3. both feet on bosu ball, for 2 min

**Standing unsupported**	Standing stable without holding.	1. feet together for 1 min on the floor<br>2. feet together for 1 min on an Airex balance pad<br>3. feet together + eyes closed for 1 min on the floor<br>4. feet together + eyes closed for 1 min on an Airex balance pad
**Tandem stance/gait**	Tandem stance: Place one foot directly in front of the other. If you feel you cannot place your foot directly in front, try to step far enough ahead that the heel of your forward foot is ahead of the toes of the other foot.<br>Tandem gait: walk in a straight line while the toes of your back foot touch the heel of the front foot at each step.	1. tandem stand as long as possible, max 2min (2*left in front of right, 2*right in front of left)<br>2. tandem gait forwards for 2 min<br>3. tandem gait backwards for 1 min
**Standing on one leg**	Stand on one leg as long as you can without holding.	1. on less impaired leg (3*)<br>2. on most impaired leg (3*)<br>3. on less impaired leg with eyes closed (3*)<br>4. on most impaired leg with eyes closed (3*)
**Picking up object from the floor**	From standing position: pick up a beanbag which is placed in front of your feet.<br>During walking: walk and pick up the bean bags from the floor (every 3m).	1. from standing position, for 1min<br>2. during walking, for 1min<br>3. during walking, for 2min
**Tapping the ground**	In standing position: tap the ground lightly with the ball of your foot.	1. with less impaired leg, on the spot next to the other foot, for 1min<br>2. with most impaired leg, on the spot next to the other foot, for 1min<br>3. with less affected leg, forwards-backwards (± 20cm for other foot, not tandem), 2*1min<br>4. with most affected leg, forwards-backwards (± 20cm for other foot, not tandem), 2*1min
**Tapping a step**	In standing position: tap lightly on a step (± 15cm height) with your foot.	1. with the less impaired leg, for 1min<br>2. with the most impaired leg, for 1min<br>3. with the less impaired leg, for 2min<br>4. with the most impaired leg, for 2min
**Gait with shoving obstacles**	Begin walking at your normal speed. When you come to the first wooden block, shove it over the line with your right leg (±10cm). When you come to the second wooden block, shove it over the line with your left leg, and so on, alternately right/left foot and medial/lateral side of the feet.	1. straight line, every 3m a wooden block (length) with a width of 1m between left and right blocks, for 2 min<br>2. straight line, every 1m a wooden block (length) with a width of 1m between left and right blocks, for 2 min<br>3. straight line, variable length (1,2,3m) between the wooden blocks with a width of 1m between left and right blocks, for 2 min
**Hopping**	Hopping on one leg.	1. on the spot, with the less impaired leg, 5*<br>2. on the spot, with the most impaired leg, 5*<br>3. on the spot, with the less impaired leg,10*<br>4. on the spot, with the most impaired leg, 10*
**Sit-to-stand**	Standing up and sitting down from a chair.	1. allowed to use their arms to push off from the chair with armrests, for 1min<br>2. allowed to use their arms to push off from the chair with armrests, for 2min<br>3. without push off from armrests, for 2min
**Stairs (15-16 steps)**	Walk up these stairs. At the top, turn around and walk down.	1. with rail uses, 2*<br>2. without rail uses, 2*<br>3. without rail uses, 4*

Table S2; Exercises during DTT.

Exercise	Cognitive function	Exercise description / Difficulty levels
**Auditory Exercises combined with walking**		
**Noise**	Auditory discrimination<br>Working memory<br>Selective attention	'Remember and recognize by saying 'yes':'<br>1. 2 particular sounds among different sounds/noises.<br>2. 3 particular sounds among different sounds/noises.<br>3. 4 particular sounds among different sounds/noises.
**Words**	Working memory<br>Verbal fluency	'Formulate a new word that starts with:'<br>1. the last letter of the word that is given within 10 sec.<br>2. the 2nd letter of the word that is given within 15 sec.<br>3. the 4th letter of the word that is given within 20 sec.
**Apple**	Working memory<br>Selective attention,<br>Auditory discrimination	'Remember and recognize by saying 'yes':'<br>1. 1 target word among other words that are semantically different.<br>2. 1 target word among other words that can be from the same category or semantically different.<br>3. 2 target words among other words that can be from the same category or semantically different.
**Reverse**	Working memory	1. Spell a 3- or 4-letter word in reverse within 10 sec.<br>2. Spell a 5- or 6-letter word in reverse within 20 sec.<br>3. Spell a 7+ letter word in reverse within 30 sec.
**Listen**	Auditory memory<br>(recognition)	'Tell whether you already heard or not heard the word that is given (heard, not heard):'<br>1. 30 words are given (at a presenting rate of 6 sec).<br>2. 35 words are given (at a presenting rate of 6 sec).<br>3. 40 words are given (at a presenting rate of 6 sec). Additionally, count how many times a specific target word is played.
**Taboo**	Verbal fluency<br>Executive function	'Describe the word you hear, without 1) using the word or parts of it, 2) use words that are derived from it, 3) gestured and noises and 4) abbreviations, initials or clues as 'sound as', 'rhymes like':'<br>1. Describe the given word in 20 sec.<br>2. Describe the given word in 30 sec without using the taboo word you will hear.<br>3. Describe the given word in 40 sec without using the 3 taboo words you will hear.
**Story**	Text comprehension<br>Verbal memory<br>Sustained attention	'You will hear a story while walking, after the story is completed answer:'<br>1. 3 multiple-choice (3 options) questions about the story.<br>2. 3 multiple-choice (4 simple options) questions about the story.<br>3. 3 multiple-choice (4 difficult options) questions about the story.
**Visual Exercises combined with stepping on**		
**Differences**	Visual discrimination	'Tell whether two images are the same or different:'<br>1. within 15 sec, the images can contain more than one difference.<br>2. within 20 sec, the images can contain one difference.<br>3. within 30 sec, the images can contain one small difference
**See**	Visual memory<br>(recognition)	'Memorize the smiley you see and choose from:'<br>1. 3 clearly different smileys which one you just saw within 10 sec.<br>2. 3 smileys with two smaller differences which one you saw within 15 sec.<br>3. 3 smileys with one small difference which one you saw within 20 sec.
**Think**	Verbal and visual<br>analog reasoning<br>Executive function	1. You see 2 series of 4 images, match the images of the 2 series that below to each other (e.g. a ring and a hand).<br>2. You see a calculation assignment on the screen and need to tell the correct answer (e.g. what letter do you get when you do K + 2?).<br>3. You see a series of symbols and one question mark, which of the 4 symbols below needs to be placed on the spot of the question mark?
**Roadmap**	Visual spatial planning<br>Mental rotation.	'You see a roadmap and the place you have to go to. Tell at each intersection which direction you want to go (forward, backward, left, right):'<br>1. Simple roadmap with a blue icon indicating your orientation.<br>2. Roadmap with roundabouts, houses and trees with a blue circle without orientation.<br>3. Complex roadmap with one-way streets, roundabouts, houses and trees with a blue circle without orientation.

Table S3; Means and mixed model analysis of cognitive DTC and correct answers (#n) in 'Digit Span' conditions.

Condition	Group	PRE Mean ± SD	POST Mean ± SD	FU Mean ± SD	Mixed model analysis *p*-values Time	Group	Time*Group	Multiple comparisons *p*-values		
**Single Digit-Span** #n Correct	DTT	6.5 ± 2.9 (n = 20)	7.3 ± 3.2 (n = 17)	7.6 ± 2.7 (n = 20)	.044*	.238	.746	PRE-POST .297	PRE-FU .013$	POST-FU .158
	SMT	7.7 ± 2.6 (n = 19)	7.8 ± 2.5 (n = 19)	8.5 ± 2.3 (n = 19)						
**Walk – Digit-Span**										
#n Correct	DTT	5.8 ± 2.6 (n = 20)	6.9 ± 2.9 (n = 17)	6.9 ± 3.0 (n = 20)	.359	.348	.202			
	SMT	7.3 ± 2.8 (n = 19)	7.3 ± 2.1 (n = 19)	7.1 ± 2.6 (n = 19)						
$DTC_{cognitive}$ (%)	DTT	7.9 ± 33.7 (n = 19)	3.7 ± 25.4 (n = 17)	10.7 ± 23.6 (n = 20)	.350	.858	.679			
	SMT	0.7 ± 38.3 (n = 19)	4.1 ± 24.8 (n = 19)	15.5 ± 25.6 (n = 19)						
**Cup – Digit-Span**										
#n Correct	DTT	6.3 ± 2.7 (n = 19)	6.6 ± 3.4 (n = 17)	7.0 ± 3.1 (n = 20)	.261	.322	.781			
	SMT	7.1 ± 2.7 (n = 19)	7.6 ± 2.0 (n = 19)	7.6 ± 2.4 (n = 19)						
$DTC_{cognitive}$ (%)	DTT	-4.3 ± 55.8 (n = 18)	8.8 ± 39.4 (n = 17)	10.7 ± 27.0 (n = 20)	.574	.808	.490			
	SMT	5.7 ± 26.7 (n = 19)	-2.0 ± 23.9 (n = 19)	8.8 ± 25.8 (n = 19)						
**Obstacles – Digit-Span**										
#n Correct	DTT	5.3 ± 2.3 (n = 20)	6.2 ± 2.9 (n = 17)	6.8 ± 2.4 (n = 19)	.003*	.163	.812	PRE-POST .036	PRE-FU < .001$	POST-FU .198
	SMT	6.6 ± 2.4 (n = 18)	7.2 ± 3.1 (n = 19)	7.6 ± 2.4 (n = 19)						
$DTC_{cognitive}$ (%)	DTT	16.9 ± 24.4 (n = 19)	13.2 ± 39.1 (n = 17)	8.9 ± 19.2 (n = 19)	.688	.503	.836			
	SMT	12.0 ± 22.3 (n = 18)	7.1 ± 32.7 (n = 19)	9.8 ± 20.3 (n = 19)						

Significant at < 0.05 (*), significant post-hoc ($). *Abbreviations:* DTT: Dual Task Training; SMT: Single Mobility Training; DTC: dual task cost; FU; follow-up; n: number; SD: standard deviation.

Table S4; Means and mixed model analysis of cognitive DTC and correct answers (#n) in 'Subtraction' conditions.

Condition	Group	PRE Mean ± SD	POST Mean ± SD	FU Mean ± SD	Mixed model analysis *p*-values: Time	Group	Time*Group	Multiple comparisons *p*-values		
**Single Subtraction** #n Correct	DTT	14.6 ± 8.1 (n = 20)	16.6 ± 8.4 (n = 18)	16.7 ± 8.9 (n = 20)	.012*	.730	.965	PRE-POST .037	PRE-FU .004$^{\$}$	POST-FU .431
	SMT	13.7 ± 7.1 (n = 20)	15.3 ± 8.4 (n = 20)	16.1 ± 8.0 (n = 20)						
**Walk – Subtraction**										
#n Correct	DTT	12.6 ± 7.1 (n = 20)	15.8 ± 8.1 (n = 18)	15.2 ± 7.2 (n = 20)	<.0001*	.804	.294	PRE-POST <.001$^{\$}$	PRE-FU <.001$^{\$}$	POST-FU .468
	SMT	11.9 ± 5.9 (n = 19)	14.4 ± 6.5 (n = 20)	15.8 ± 6.8 (n = 20)						
$DTC_{cognitive}$ (%)	DTT	5.1 ± 41.9 (n = 20)	1.9 ± 24.6 (n = 18)	1.3 ± 33.8 (n = 20)	.339	.501	.599			
	SMT	4.7 ± 26.4 (n = 19)	-2.5 ± 28.2 (n = 20)	-13.3 ± 62.6 (n = 20)						
**Cup – Subtraction**										
#n Correct	DTT	12.0 ± 7.3 (n = 20)	14.2 ± 6.5 (n =18)	14.3 ± 6.9 (n = 20)	.013*	.758	.766	PRE-POST .008$^{\$}$	PRE-FU .016$^{\$}$	POST-FU .750
	SMT	13.1 ± 7.4 (n = 20)	14.9 ± 6.4 (n = 20)	14.3 ± 7.2 (n = 20)						
$DTC_{cognitive}$ (%)	DTT	17.3 ± 24.5 (n = 20)	10.0 ± 31.3 (n = 18)	7.9 ± 32.9 (n = 20)	.560	.101	.517			
	SMT	-2.9 ± 46.5 (n = 20)	-9.2 ± 36.6 (n = 20)	1.9 ± 45.1 (n = 20)						
**Obstacles – Subtraction**										
#n Correct	DTT	12.8 ± 7.1 (n = 20)	15.1 ± 8.2 (n = 18)	14.4 ± 7.1 (n = 20)	.057	.767	.830			
	SMT	12.6 ± 6.6 (n= 20)	13.7 ± 6.4 (n = 20)	13.9 ± 6.4 (n = 20)						
$DTC_{cognitive}$ (%)	DTT	3.0 ± 42.9 (n = 20)	7.1 ± 33.2 (n = 18)	4.5 ± 43.9 (n = 20)	.450	.732	.649			
	SMT	-7.9 ± 66.5 (n = 20)	3.5 ± 37.9 (n = 20)	7.1 ± 39.9 (n = 20)						

Significant at < 0.05 (*), significant post-hoc ($^{\$}$). *Abbreviations:* DTT: Dual Task Training; SMT: Single Mobility Training; DTC: dual task cost; FU; follow-up; n: number; SD: standard deviation.

Table S5; Means and statistical analysis of cognitive DTC and correct answers (#n) in 'Vigilance' conditions.

Condition	Group	PRE<br>Mean ± SD	POST<br>Mean ± SD	FU<br>Mean ± SD	Within groups *p*-values<br>PRE-POST	 PRE-FU	 POST-FU	Between groups per time *p*-values<br>PRE	 POST	 FU
**Single Vigilance**<br>#n Correct	DTT	23.7 ± 0.7<br>(n = 19)	23.6 ± 0.7<br>(n = 18)	23.8 ± 0.7<br>(n = 20)	.713	.783	.157	.835	.654	.820
	SMT	23.7 ± 0.7<br>(n = 20)	23.8 ± 0.6<br>(n = 20)	23.8 ± 0.5<br>(n = 20)	.773	.581	.792			
**Walk – Vigilance**										
#n Correct	DTT	23.4 ± 0.9<br>(n = 20)	23.7 ± 0.8<br>(n = 18)	23.9 ± 0.3<br>(n = 20)	.196	.031	.480	.758	.534	.398
	SMT	23.5 ± 1.1<br>(n = 20)	23.7 ± 0.6<br>(n = 20)	23.6 ± 1.0<br>(n = 20)	.557	.831	527			
$DTC_{cognitive}$ (%)	DTT	1.5 ± 4.3<br>(n = 19)	-0.5 ± 3.3<br>(n = 18)	-0.5 ± 3.6<br>(n = 20)	.212	.284	.308	.945	.534	.429
	SMT	1.1 ± 3.5<br>(n = 20)	0.4 ± 3.4<br>(n = 20)	1.0 ± 4.4<br>(n = 20)	.440	.766	.535			
**Cup – Vigilance**										
#n Correct	DTT	23.1 ± 1.4<br>(n = 20)	23.7 ± 0.5<br>(n = 18)	23.7 ± 0.6<br>(n = 20)	.088	.046	1.000	.192	.141	.698
	SMT	23.6 ± 0.7<br>(n = 20)	24.0 ± 0.2<br>(n = 20)	23.6 ± 0.8<br>(n = 20)	.053	.794	.023			
$DTC_{cognitive}$ (%)	DTT	2.3 ± 6.8<br>(n = 19)	-0.3 ± 3.2<br>(n = 18)	0.3 ± 3.5<br>(n = 20)	.320	.500	.366	.270	.553	.583
	SMT	0.4 ± 3.9<br>(n = 20)	-0.9 ± 2.7<br>(n = 20)	1.0 ± 3.9<br>(n = 20)	.326	.554	.128			
**Obstacles – Vigilance**										
#n Correct	DTT	23.1 ± 1.2<br>(n = 20)	23.6 ± 0.6<br>(n = 18)	23.7 ± 0.7<br>(n = 20)	.032	.046	.429	.883	.675	.478
	SMT	23.2 ± 1.0<br>(n = 20)	23.6 ± 0.8<br>(n = 20)	23.7 ± 0.5<br>(n = 20)	.163	.053	.763			
$DTC_{cognitive}$ (%)	DTT	2.6 ± 5.8<br>(n = 19)	0.2 ± 3.5<br>(n = 18)	0.4 ± 3.7<br>(n = 20)	.218	.281	.553	.989	.988	.640
	SMT	2.0 ± 4.7<br>(n = 20)	0.6 ± 4.2<br>(n = 20)	0.6 ± 2.5<br>(n = 20)	.293	.337	.972			

Significant multiple comparisons ($^{\$}$). *Abbreviations:* DTT: Dual Task Training; SMT: Single Mobility Training; DTC: dual task cost; FU; follow-up; n: number; SD: standard deviation.
